# Online learning during the COVID-19 pandemic: A qualitative study among final year medical students at the University of Zambia

**DOI:** 10.12688/f1000research.124823.1

**Published:** 2022-11-22

**Authors:** Anthony Nsamba Limbumbu, Jane Chanda Kabwe, Andrew Kumwenda, Phyllis Chibuye Kasonkomona, Grace Mwila, Mwansa Ketty Lubeya

**Affiliations:** 1 Young Emerging Scientists Zambia, Lusaka, Lusaka, 10101, Zambia; 2 Department of Anaesthesia and Critical Care, National Heart Hospital, Chongwe, Lusaka, 10101, Zambia; 3 Women and Newborn Hospital, University Teaching Hospitals, Lusaka, Lusaka, 10101, Zambia; 4 Department of Obstetrics and Gynaecology, The University of Zambia-School of Medicine, Lusaka, Lusaka, 10101, Zambia; 5 Development Division, Technical Education, Vocational and Entrepreneurship Training Authority, Lusaka, Lusaka, 10101, Zambia; 6 Graduate women Zambia, Lusaka, Lusaka, 10101, Zambia

**Keywords:** virtual learning, final year medical student, medical education, COVID-19 pandemic, quarantine

## Abstract

Background

Since the globe was faced with the COVID-19 pandemic in December 2019, numerous adjustments have been made in all sectors to curtail the spread of infection. Most elementary and tertiary schools were closed or suspended until the transmission rates dropped. Following the outbreak of COVID-19, medical schools in Zambia have sought ways to replace face-to-face medical learning with virtual clinical teaching.

The objectives of this study were to explore the perceptions of online learning among University of Zambia medical students and understand the barriers and facilitators to effective online learning.

Methods

A qualitative descriptive approach was used, enrolling final year medical students from the University of Zambia; the consenting participants were sampled purposively and interviewed through virtual platforms until data saturation was reached upon interviewing the 11 ^th^ participant. A total of 14 participants were interviewed, audio recorded, transcribed verbatim and data was analyzed using six steps of thematic analysis.

Results

Three broad themes arose from the interviews: online learning perceptions, facilitators and barriers to online learning. Regarding perceptions of online learning, they highlighted that the delivery was simple to understand, with convenient scheduling and the benefit of being able to refer back to the recorded lectures. Some barriers encountered during the online learning were poor network connection, frequent power outages, lack of patient-student interaction and challenges with learning space in their homes. The facilitators were self-paced learning, availability of lecturers and the desire to complete their training despite the lockdown being in effect.

Conclusions

Most medical students had positive perceptions of online learning despite its challenges. With the improvement in technology, online education should be incorporated into the traditional training of medical students to get the best outcomes.

## Introduction

With the rise of the COVID-19 global pandemic, many learning institutions across the world had to shut down to slow the spread of the disease; an estimate of at least 1.5 billion children and youth were affected by the closures. ^1^ To minimize interference in academic activities, online teaching and learning platforms were introduced in several countries with high levels of digital penetration. ^2^ To curb the spread of the COVID-19 pandemic, the government in Zambia, through the Higher Education Authority, closed all the institutions. This prompted the University of Zambia (UNZA) to employ online teaching and learning via Moodle and ASTRIA to mitigate the impact of COVID-19 on academic activities. ^3^ Moodle is a widely used, open-source learning platform that comes without a cost and enables educators to create interactive online courses. At the same time, ASTRIA is a paid service that provides several different management systems for the students and educators. ^4^ Some lecturers used other platforms such as ZOOM, Google Meet and WhatsApp according to their preferences. This transition was made for all the programs offered at UNZA, including the Bachelor of Medicine and Surgery.

Online learning, also called virtual or e-learning, is the acquisition of knowledge through digital technologies and media, typically by accessing the internet or any electronically enabled learning. ^5^ Online learning is variable among medical schools, and is more common in basic sciences than in clinical clerkship years. In clinical years, however, it has been used to increase the effectiveness of conventional face-to-face methods as it has its limitations. ^6^ The discipline of medicine is about saving lives whilst upholding patient safety, and any significant alterations in how it is taught call for intensive consideration. Most of the activities and teaching in medical training are practical-based ^7^ and require physical interaction with the patients, laboratories and theatres. This underscores the importance of social interactions in the constructivist learning theory, which is the philosophical underpinning principle of experiential learning. ^8^ The UNZA had never taught medical students on a full-time digital platform. This meant that students needed to adapt to the new mandatory digital methods, including final year medical students who were soon to be interns and attending to patients as the first health professionals the patients would encounter in many hospitals in the country. Online teaching and learning were introduced at a time when the country was faced with challenges regarding consistent electricity supply countrywide. Some communities faced up to 15 hours of load shedding per day of electricity, with some parts of the country, such as rural areas, experiencing poor internet connectivity. ^9^ During load shedding periods, the affected areas had no electricity supply from the country’s only power supply institution, meaning a total blackout.

In an integrative review conducted by O’Doherty *et al.,* prior studies highlighted significant barriers to online learning that include: time constraints, attributed lack of incentives to engage in online education, deficits in technical skills, inadequate infrastructure like technology and quality of services, lack of institutional strategies and support as well as negative attitudes of the people involved. ^10^ Some of the suggested solutions to these identified barriers include adopting digital tools that could free up some time, collaborating with relevant stakeholders and departments with the adoption of new approaches, as well as maintaining a positive attitude among all team players.

This study aimed to explore the experiences of final year UNZA medical students as they transitioned to online learning to complete their medical training program and highlight the barriers and facilitators.

## Methods

We used a descriptive qualitative approach to understand the experiences, barriers and facilitators of online learning among final year medical students for 2020 at the UNZA during the COVID-19 pandemic lockdown. Thus this study was imbedded within the interpretivism paradigm in trying to explore the perceived barriers and facilitators for the students’ online learning during the lockdown as the underlying philosophical approach because reality is deemed to be socially constructed. ^11^ Since online learning is a fairly new concept in this setting, we adopted the individual facet of technology acceptance model (TAM) which has been used in other studies to show user acceptance of the technology based on the perceived usefulness and perceived ease of use. ^12^

### Ethics and consent

Ethics approval for this study was obtained from the University of Zambia School of Medicine Research Ethics committee (UNZASOMREC) on the 27 ^th^ of July 2020 with Assurance No. *FWA00000338* and the Dean of Students Affairs (DOSA) Ridgeway campus. Written informed consent was obtained from the students that participated in the study.

### Participants

The target population was the final year medical students that were being trained at the UNZA during the time of the lockdown. A purposive sampling method was used with the criteria that the participants had to be in final year, at the UNZA and had participated in online learning at the time of selection. The participants were chosen irrespective of their residence of origin and were picked until saturation of data was achieved. This was considered attained upon interviewing 11 participants, however we interviewed three more participants making the total number of study participants 14. Data saturation is when enough data has been collected to replicate the findings, reached in this study after interviewing 11 participants. ^13^ No participants refused to participate nor dropped out of this study.

### Procedure

Written informed consent was obtained from each participant. The participants signed the informed consent virtually which was sent and received back in PDF format through WhatsApp messenger. The data was then collected through scheduled in-depth interviews via the Zoom online platform and direct phone calls to minimize physical interactions with the participants. Three interviews were conducted by a medical student, male ANL together with an Obstetrics and Gynaecology specialist, female doctor MKL who is the corresponding author with several years of experience in research. The remaining 11 interviews were conducted by ANL supervised by MKL. This ensured that there was no power differential between the respondents and the researcher as it is easier to create a good research environment with a colleague and not withhold any information back for the purpose of the research. The interviews were conducted with videos turned off to facilitate good connectivity and ensure confidentiality as the interviews were being recorded. Other researchers have found the Zoom platform to be a good tool for collecting qualitative data because it is easy to use, cost-effective, and has good data management features and security options compared to other interviewing mediums like direct telephone and face-to-face. ^14^ Telephone calls were done for two participants as they had poor internet connectivity in their residences. Interviews were conducted in English, audio-recorded and transcribed verbatim. The transcripts were returned to the participants to ensure there was no loss in information from audios, however three participants did not respond. The duration of each interview was 19–47 minutes.

### Materials

We used an in-depth interview guide that was pre-tested and adjusted for clarity and ease of understanding; The questions were paraphrased in a simplified manner, ensuring the words were not too technical for the study participants. To avoid ambiguity, the questions were made to be concise, specific and relevant to the information required. The interview guide comprised open-ended questions with follow-up probes in instances where certain information was left out. ^15^ Transcripts were then coded in preparation for analysis.

### Analysis

The data was coded through inductive coding approach and we employed the six steps of thematic analysis, a practical data analysis method used in qualitative research. It was advantageous since we explored participants’ experiences; it enabled us to develop a structured approach. ^16^ Three researchers (JCK, AK, MKL) coded the transcripts based on the emerging themes from the respondents. We did not use any qualitative data analysis software during the analysis. All six researchers analyzed the data based on the codes generated and they expressed reflexivity throughout the entire process of this study.

## Results

The demographic characteristics of the participants are presented in Table 1 below.

**Table 1.** Demographic characteristics of participants.

ID	Age	Sex	Marital status	Province - City
p1	27	Male	single	Copperbelt -Kitwe
p2	26	Female	single	Eastern - Chipata
p3	24	Male	single	Lusaka - Lusaka
p4	25	Male	single	Lusaka - Lusaka
p5	26	Female	single	Southern - Monze
p6	27	Male	single	Lusaka - Lusaka
p7	24	Male	single	Central - Kabwe
p8	25	Female	single	Lusaka - Lusaka
p9	27	Male	single	Copperbelt - Kitwe
p10	26	Male	single	Copperbelt - Ndola
p11	26	Male	single	Copperbelt - Mufulira
p12	27	Female	single	Lusaka – Lusaka
p13	29	Male	single	Lusaka – Lusaka
p14	28	Male	single	Lusaka – Lusaka

The ages varied between 24 and 29, with all participants being single and unemployed. There were four female and ten male participants. The participants represented five of Zambia's ten provinces, including Copperbelt, Eastern, Central, Southern and Lusaka.

We considered the broad themes that affected the effectiveness of online learning during the COVID-19 lockdown: online learning perceptions, barriers and facilitators to online learning. Under these themes, we established sub-themes to provide a deeper understanding. Table 2 below shows the summary of the themes.

**Table 2.** Thematic presentation of the participant’s experiences and perception of online learning.

	Broad theme	Sub-theme	Description
1.	**Online learning perceptions**	Simplicity	The ease and convenience with which materials are delivered and absorbed
		Scheduling of lectures	Any systematic, orderly way of delivering the course work
		Self-directed learning	Any action taken by individual to facilitate learning
2.	**Barriers**	Quality of internet and access	Limitations with online learning arising from challenges with availability and reliability of data bundles
		Power outages	Lack of electricity to charge gadgets during online learning
		Lack of patient-student interaction	The process of learning not allowing real time, face to face interaction and engagement between students and patients
		Lack of learning space at home	Not having a conducive learning environment
3.	**Facilitators**	Self-paced learning schedules	Learning that can be accessed at any time convenient to the students
		Support systems (family, friends, University)	Any person or process which facilitates the process of learning
		Prospects of completing school	Intrinsic motivation based on final goal of the student such as graduating

**Theme 1: Online learning perceptions**

The participants' perceptions are discussed under three sub-themes: simplicity, scheduling, and self-directed learning or skills development. Overall, the participants' perceptions of online learning were generally positive, as some expressed their approval of this method, commending the University’s initiative of switching to online learning during the lockdown to facilitate the continuity of their studies.

***Simplicity***

The first sub-theme we considered under this core theme was simplicity, where most participants stated to have been able to understand the presentations delivered as both lectures and notes with ease. When asked what they thought about the material that was provided online, one participant said:

*“Oh yeah, it was excellent. They (lectures) were readily available especially for the paediatrics course when they teach; they would make sure they upload, and also for the previous courses that I did they would make sure they upload all the lectures that they had uh done…” (p4)*

Not only was the material said to have been uploaded for easy accessibility, but the content was also excellent and sufficient according to the requirements of the students, as stated by participant 8 quoted below:

*“They (online lectures) were something new firstly, but uhm they were detailed, uh the doctors tried and were able to explain their material adequately, so they were great.” (p8)*

However, not all participants were satisfied by the lecture notes provided online; some expressed their disapproval of online learning, implicitly stating the need for further explanation of the lecture notes administered to them:

*“Uhm, the material was okay … it had enough content just that it lacked explanation, you know, it was just given (…) and (…)I had to read on my own, but the content was enough though you need an explanation to get something uh to a 100% uh level of understanding”*

*(p11)*

***Scheduling***

The lessons were delivered by sending lecture notes to the students to study at their convenience, and then they were assessed on this material. The assessment was done physically following the uploading of notes, thus combining traditional ways of evaluation with virtual methods of teaching. This was attested by the response below:

*“I think they tried their best as well; they sent notes like every- for everything on their lecture schedule was sent, they sent clinical scenarios, and they had to give us a physical test before the exams, so they did their best as well (…)”*

*(p11)*

Lectures were also delivered online in real-time, with interactions between the students and lecturers made possible by the Zoom platform.

*“Well, uh, we had lectures for about an hour in the morning, and an hour in the evening. And of course, with different doctors coming in and try to help out.” (p3)*

*“(…)the fact that most of the lecturers were willing to let us ask questions, for me that was the major thing”*

*(p12)*

Furthermore, as part of the training, the medical students were also assessed online through an assignment that required to be downloaded, answered and uploaded to complete the task.

*“The assignment was sent on Moodle (online learning platform), and we had to download, then we answer in a word document, then you send it back via Moodle (…)”(p2)*

***Self-directed learning***

Self-directed learning (SDL), a strategy in which learners take responsibility for their learning, was yet another sub-theme that emerged, highlighting the participants’ willingness to augment their online learning. With medical training being a hands-on program, students need to learn certain skills to aid in patient management. A couple of examples are highlighted below how students were able to use their initiative to facilitate their learning:

*“so we had to make sure that we learnt the practical aspect as well as helping the patient on the ground and lessening the work of the doctors in our units and being available as always to follow up certain things in case uh one or 2 doctors were busy, yes, not forgetting the nurses as well…” (p6)*

*“the day before or two days before our ward rounds we would clerk our patients then we would just come and present so that we facilitate the learning as we are presenting …”*

*(p12)*

**Theme 2: Barriers**

Online learning had challenges; students and lecturers faced various difficulties with this method of sharing knowledge, such as the inability to navigate around the platform. We grouped the barriers encountered by the participants into four sub-themes: quality of internet and access, power outages, lack of patient-student interaction and lack of learning space at home. These barriers were beyond the students’ control and could not be easily overcome.

***Quality of internet and access***

Internet connectivity in the country depends on the network provider one subscribes to and the geographical location. The most prominent of these factors is the geographical location, where certain areas of the country struggle with internet connectivity more than others. Moreover, one needs to pay for internet access by buying data bundles to enable them to access online platforms. The University of Zambia offers internet access within the school premises through Wi-Fi routers; however, with the school’s closure, the students had to fend for themselves to have internet access because they were no longer on campus.

*“…At times you would find that the network is not good, so you are not able to get the lecturer clearly”* *(p13)*

*“…I remember one time what happened I was just blocked out like network couldn’t log on, bundles were there I just couldn’t connect, so there is the issue of network …” (p3)*

While others struggled with internet connectivity, a few had challenges with access to the internet as they occasionally could not afford to purchase data bundles. These students would then be forced to abscond classes and either watch the lecture video if it was recorded or just read the lecture notes if and when they are made available. Some participants stated not to have always been able to afford data bundles to access online lectures.

*“Then sometimes there were issues of finances where you don’t have enough money for bundles (…)” (p2)*

*“And also issues of finances coz we were expected to be spending a lot of money for us to be managing to learn online, so it was a bit difficult, so it set me back and when it came to catching up on things and understanding things it took an extra effort for me to do so.” (p4)*

***Power outages***

Zambia was experiencing load shedding in many regions to prevent excessive load on the main generating electric plant. With specific areas experiencing electricity power cuts for as long as 8 hours daily, such were the conditions that the final year medical students had to learn under as they were not exempted from these power cuts.

*“(…) Number two, we have what we call power cutting schedule, so sometimes they would put up lectures during the time that there is no power you should not- you haven’t charged your phone you would not be able to attend, so it was a setback.” (p4)*

*“Then, of course, we also missed on several lectures, and as well as when online learning started (…) sometimes we would have load shedding where you are and all those things. Yeah.” (p2)*

*“I think the issue may be that I had was maybe was with the load shedding (…) cause sometimes I would struggle to have a few of the lectures we were having online on the Zoom platform because of the load shedding issues that we would have from time to time (…)”* *(p14)*

***Lack of patient-student interaction***

Traditionally, clinical students learn most of their skills in the hospital wards, where they can interact with patients. Through these interactions, medical students are therefore able to practice therapeutic and diagnostic procedures as well as counselling patients. Virtual education resulted in the cessation of student-patient interactions, which affected the students.

*“Well, uh, you can’t learn medicine online, I think that’s my perception about it you can't do a lumbar puncture online”* *(p11)*

With medical training being a hands-on program, the best place to train medical students remains at the patient’s bedside; despite being able to read various textbooks and presentations, a medical student's skills are best developed at the bedside through practical participation. Students need to learn specific skills to aid in patient management. The impact on the acquisition of skills that the lack of patient-student interaction brought was highlighted as follows:

*“It shattered my dreams of acquiring the basic skills that I needed to acquire, the things that I thought I would do in 7th year (referring to the final year of undergraduate medical training) that I didn't do in 5th year I didn't do in 6th year so I thought I would perfect on certain things in 7th year yeah so it shattered a lot on my side,”* *(p11)*

*“For clinical students, I don’t think like online learning is enough you still need to have the practical part of it (… ) coz like they say diseases don't read textbooks so the textbook can explain to you as proper as possible, but when you go on the ward it will still look like something new something you had never heard before when you learnt about it (…) the negative part is that you don’t have the practical skills like for you to be able to see that on a patient, for you to be able to apply your knowledge, the application part of it lacks so I feel like yeah it’s okay, but I don’t think like it’s very appropriate or good enough for a clinical student” (p5)*

To balance the practical aspect of the training with the theoretical one, some participants proposed methods in which students could do their theory work online and still find time to go to the ward for their practical training or even make use of the skills lab:

*“I think it can be a very positive thing to have lectures online, uh through Zoom like this discussion but still be expected to go to the ward for the practical sessions.” (p6)*

*“At least if a way to keep us in school was found and a way of opening up maybe a skills laboratory so that in groups of small individuals with one or two lecturers (… ) you can now go and perform some of the things that you learnt online imagine you are being taught how to catheterize, how to cannulate I think it can make actually uh a good idea if students learnt in their hostel online doing their learning then afterwards have some time to perfect their skills (okay), even just for an hour, yes.”* *(p11)*

***Lack of learning space at home***

In most homes, dependents are expected to help their guardians with chores that are usually routine and executed at specific time frames. With the medical students being sent home, they had to perform these chores as per custom. Furthermore, some houses’ setups have noisy environments or are too crowded to be impeccable for studying or attending online lectures. Moreover, none of them had designated places to enable participants adequately attend to their academic responsibilities. This was considered to be interfering with the learning process of some of the participants.

*“I had to factor those things chores, noise at home just it wasn’t a favourable environment, it wasn’t a favourable environment, so this is where you are sharing a bedroom, you can’t lock yourself up so such things had to come in and it was it was a huge challenge in terms of the way I live as an individual here (school)”* *(p11)*

Learning from home proved to be a barrier for most of the participants. When asked how the living arrangements fitted in with online learning, p3 and p12 had this to say:

*“Okay, uh, when we were back in school and learning from school, the living arrangements were okay, I would say okay, but then being home, coz at home you are not exactly a student. At home, you are a child, so you have to help out here and there, so it becomes very challenging cause one moment, of course, you are supposed to have classes at 9 (am), but then the parents want you to help out with something, so I found that challenging.” (p3)*

*“They don’t fit at all. You know when I'm home, it’s a tiny place, so everyone is everywhere. I would inform them that I had class, and I would sit in the bedroom so that I would have some privacy, but everyone would come in, they’ll start calling me, and I had to remind them I was actually in class; they want to talk to me there and then and I am like, okay you people I am in class now. It's tough to even study from home, everyone expects you to work, prepare lunch, do the house chores, and then I have no time for my books because, by the time I finish everything, I am exhausted. I cannot touch a book, and you know how med school works like it requires a lot of time accorded to it to understand a certain concept, so home is not conducive at all.”* *(p12)*

**Theme 3: Facilitators**

Three sub-themes emerged from the final core theme considered: self-paced learning schedules, support systems and prospects of completing school. These sub-themes expressed the factors that helped enhance the students’ learning experience.

***Self-paced learning schedules***

All participants regarded online learning as advantageous because they could easily refer back to the content given either during recorded lectures or in PowerPoint presentations. The participants revealed the following:

*“So, I think one thing I found interesting was that some of the lectures were recorded, meaning if you missed a lecture, you could easily just download and go through the lecture on your own time. Then the other good part was that since you have the downloaded lecture, meaning you can go through the lecture over and over so that you understand better, yes, so I think those were the positives.” (p2)*

*“Okay, uhm, my experience was good, yes it was very good I was able to understand what the lecturer was saying … and uh and at least it was recorded, so I had a chance to be going through over and over again, it was a good experience” (p4)*

*“So, what worked well for me was uh going back to the recorded uh lectures, yes it took a lot of time but at least it would help me understand what the uh lecturer was trying to tell me and also the fact that you know how when somebody is talking it’s rapid there are certain points that you would miss out but if you go back and listen to them it will be easier” (p5)*

***Support systems***

The support systems of the students during this period were explored. Most respondents said they had not received any support from the University. In contrast, others considered the availability of lecturers during this period as a means of support from the University. Some of the participants’ verbatim regarding support from the University are highlighted below:

*“The university? Support? Uh no, not really. No, they didn’t.” (p7)*

*“Uh, well, I think they tried to push our lecturers to do their work and also we had some Moodle lectures on how best you can use Moodle for you to be able to access the online lectures” (p5)*

Others, however, allude to the support from colleagues as one of the facilitators.

*“Okay, so uh issues of uh maybe discussing, discussions, sorry, discussions with my friends online it gave room for me to get in touch with people, a good number of people from different courses and we’d hold a meeting effectively, so it was it was one good thing for me.” (p4)*

*“(…) I asked some of my friends on how to go about it, activating the audio and everything, yeah it did improve with time at least I could easily join in and I was able to get the lecturer” (p2)*

***Prospects of completing school***

Being at the verge of completing their undergraduate training and facing the closure of physical attendance of school, the medical students were fully receptive to the progression of lessons virtually.

*“I'm saying in a setting where we needed to learn and complete our studies, I think the online learning served well, yeah.” (p7)*

*“(…) I think in as much as the online thing was forced on us, it was one way of helping us graduate, help us learn and catch up. I think there was a need for this online learning to continue (…).”* *(p11)*

*“Firstly, I was able to continue my academic life despite the pandemic and complete my studies (…).” (p8)*

## Discussion

The study explored the experiences of final year medical students at the UNZA doing online learning during the COVID-19 pandemic. Online learning was delivered in a scheduled and simplified manner. However, there was unreliable internet connectivity and access, power outages and diminished patient-student interaction. Notwithstanding, positive online learning experiences were identified as self-paced learning, support systems and the thought of graduating from medical school.

The primary study findings are that their online learning was delivered in a scheduled and simplified manner, whilst they also had an element of self-directed learning. The notable barriers to this online learning were the variability in internet connectivity and access, power outages, and a lack of patient-student interaction impeding their acquisition of technical skills and a lack of learning space in their homes. On the other hand, highlighted facilitators to online learning include self-paced learning, having to support systems, and the prospects of completing medical school, which motivated them. Online learning provided the best available means for the students to continue their education during the lockdown, as the pandemic kept spreading with poor control globally, thus presenting an indefinite period of spread. ^17^

The study participants were satisfied with the transition to online learning, commended the course delivery, and felt it was simple and convenient. Earlier studies also show that online learning was welcomed as the best alternative for continued education for the students given the circumstances. ^18^ However, it was recommended that a combination of online learning and physical attendance in class should be used to maximize the benefits of education. ^19^ Contrary to this welcomed initiative, a different study reported a general dissatisfaction by the students that led to a disruption of learning. ^20^ It was also previously established that the student’s perception and ability to adapt to their learning environment were essential to achieving learning. ^21^^,^^22^

Online learning was considered a platform that allowed students to set their own pace regarding learning. Our participants stated they were able to refer back to the recordings, and they could replay them as much as they would have loved to gain a deeper understanding. This student-centered advantage of online learning was also reported by Mukhtar *et al.* in that the students learned asynchronously during the day and also remotely from various locations across the country. ^23^ Considering that it enabled these students to log in from various geographical locations, this was deemed beneficial to them as they could easily access lectures with great convenience.

One of our participants' negative learning experiences was the inability to develop skills through online learning. Participants felt that the practical aspect of the training was deprived and shattered the possibility of acquiring basic skills and needed supplementation with physical attendance to aid in skills development. Similar findings were reported in China, where some students in one study also thought it was necessary to reteach the topics physically to gain a comprehensive understanding. ^24^ Similarly, Mukhtar *et al.* in 2020 found online learning inefficient and difficult to maintain academic integrity. Skills could not be taught; there was a lack of student feedback, poor attentiveness, lack of discipline and plagiarism during assessments. ^25^

Another negative online learning experience that the students reported arose from their environment. They reported to have had challenges with internet connectivity, and the poor quality of internet access led to the students failing to log in and being logged out of lectures. This challenge was not unique to our study participants but has also been encountered by students in several other studies. ^26^^–^^28^ According to the World Bank, many African countries already have substandard internet quality of less than 10mbps. With the institution of the COVID-19 lockdown, there was increased traffic on the internet which led to slow internet speeds with reductions of as much as -10%. ^29^ Another challenge was frequent electricity power outages reported by some participants who missed some of the lessons as a result of the power challenges. Similarly, students in India also reported poor network and electricity power cuts as disturbing the online learning process. ^30^ Electricity outages were coupled with the high demand on the electricity industry encountered during the lockdown, which immediately ameliorated with the lifting of the quarantine. ^31^

Lack of learning space was yet another highlighted challenge some participants reported. There were various distractions to their learning schedule such as noise, chores and other competing demands. These findings were also reported by Ishita *et.al.* in 2021, whereby the students would encounter distractions that eventually affected their performance in exams. ^32^ Other studies found mind wandering as one of the distractions to online learning ^33^^–^^35^ but this phenomenon was not reported by any of our study participants.

Furthermore, the students indicated that the absence of interaction with patients for their training during this period was a barrier to adequate learning. For instance, one participant alluded to how he needed to be able to put the theoretical knowledge into practice and be able to appreciate the theory on an actual patient. Singh described the importance of student-patient interaction as it aids in building social intelligence, confidence, communication skills, coming up with differential diagnoses following clinical examination and how the patient is the best teacher for the student than a book would be. ^36^ Another study reports that students were not able to contribute to patient care anymore as a result of the closure. ^37^

Smith reported that some universities offered support for the students to cater for their mental health and financial support. Mental health assistance had always been there for these students, but with the arrival of the pandemic, it was now accessible through video call counselling sessions. More than half of their students had reported deterioration of their mental health during the lockdown. Although there was no financial support from the University that our participants reported, Smith reported a hardship fund that their students acquired from the institutions’ financial support services ^38^ whose idea was also recommended recently by Rainbow and Dorji. ^39^ Our participants considered the availability of lecturers and technical guidance on how to navigate the online platforms as the only support they received from the University.

### Recommendations

We recommend a blended learning approach for medical students consisting of both physical and virtual training. This will make both the lecturers and students well oriented with the use of technology and the constant feedback from the users will compel faster improvements to the virtual platform developers. Furthermore, it would be preferred for the various learning institutions to develop or improve their own virtual learning platforms which should cater for the respective needs of various programs offered by the institution. This will call for increased engagement between the University management, lecturers, information communications technology (ICT) department and student representatives. We also recommend that in instances where physical attendance of training in large groups is not feasible, as it was during this pandemic, students be allowed to do their clinical placements in smaller groups. They can also achieve this by having the students be at liberty to do the placements at any hospital convenient for them provided they are guided on the basic learning requirements in order for them to graduate. This can be done through logbooks as is the case in their primary training institutions.

### Strengths and limitations

This study endeavored to highlight the online learning experiences of final year medical students during the COVID-19 pandemic which has not been previously reported. Further, the qualitative method provides a deeper understanding of the perceptions, barriers and facilitators of online learning thus enhancing the rigor of the study. The responses were transcribed verbatim and counter-checked to verify accuracy through an iterative process that was equally applied to the generation of themes hence adding to the credibility of the study. Whilst the results are not generalizable as is the case for qualitative studies, ^40^ there is immense value in the themes identified which the University could put into consideration and inform policy when online learning is engaged for medical students as their learning needs are unique.

The limitation of this study lies in its lack of triangulation in that we only focused on one data collection tool which was the in-depth questionnaire administered to only the final year medical students from one institution and we did not use other tools such as focus group discussions (FGDs) and considered other academic years. However, with the COVID-19 restrictions, it was not feasible to have meaningful FGDs. Conducting interviews using a virtual platform without recording in order to increase the bandwidth or telephone meant we were not able to take into account the participants’ body language, which would have aided in a more comprehensive analysis. This study will have to be followed up with a quantitative study consisting of larger sample size, a validated study tool and the incorporation of different learning institutions.

## Conclusion

Despite its potential benefits, online learning cannot completely replace the traditional training of medical students. However, it could be considered supplemental to student clinical placements. As the information communications technology industry develops further, there is hope for future training to have better delivery virtually with better student satisfaction.

## Data availability

### Underlying data

Figshare: Online learning during the COVID-19 pandemic: A qualitative study among final year medical students at the University of Zambia, https://doi.org/10.6084/m9.figshare.21086992.v1. ^41^

Data are available under the terms of the Creative Commons Attribution 4.0 International license (CC-BY 4.0).
