# Supplementary material for: Online learning during the COVID-19 pandemic: A qualitative study among final year medical students at the University of Zambia
Source: F1000Res. 2022 Nov 22;11:1363. [Version 1] doi: 10.12688/f1000research.124823.1 (PMC11362714; doi:10.12688/f1000research.124823.1)
Supplement: Peer review history

# Reviewer report: Reviewer response for version 1

**DOI:** 10.5256/f1000research.137058.r244781

**Published:** 2024-08-29

**Authors:** Nirav Nimavat K

**Affiliations:** 1 Department of Community Medicine, Dr Kiran C Patel Medical College and Research Institute,, Bharuch, Gujarat, India

The qualitative study elaborates on the perceptions and barriers to medical education through an online portal.

 As this is a qualitative study, the methodology, results, and statistical analysis are different.

 The study gathers subjective feedback related to the thematic approach of the objectives.

  Points to be improved:

 1. Grammatical errors and sentence should be formed appropriately as required,

 2. The recommendation part requires modifications in terms of length. Also, recommendations should be based on the study findings.

Is the work clearly and accurately presented and does it cite the current literature?

Yes

If applicable, is the statistical analysis and its interpretation appropriate?

Not applicable

Are all the source data underlying the results available to ensure full reproducibility?

No source data required

Is the study design appropriate and is the work technically sound?

Yes

Are the conclusions drawn adequately supported by the results?

Yes

Are sufficient details of methods and analysis provided to allow replication by others?

Yes

Reviewer Expertise:

public health, epidemiology, statistics, medical education

I confirm that I have read this submission and believe that I have an appropriate level of expertise to confirm that it is of an acceptable scientific standard.

# Reviewer report: Reviewer response for version 1

**DOI:** 10.5256/f1000research.137058.r213506

**Published:** 2023-11-08

**Authors:** Forman Erwin Siagian

**Affiliations:** 1 Department of MedicineDepartment of Parasitology, Faculty of Medicine, Universitas Kristen Indonesia, East Jakarta, Special Capital Region of Jakarta, Indonesia

Limbumbu et al conducted a study regarding the effectivity of online learning exerience during COVID pandemic from the perspective of final year medical students (n=14); and even the number of participant is limited, but their qualitative approach gave us a more specific problems regarding their experiences, barriers and factors that can facilitate them to adjust or modify the problem ^1^, and this showed us the benefit of using qualitative approach in limited setting ^2^. Financial related infrastructure and temporal obstacles are always become the main problem for delivering good online teaching ^2^.

 Zambia as a country also struggling economically, and this give big impact to other sector, including medical education ^3^, ^^and this perhaps became the "silent iceberg phenomenon" that caused "poor quality internet access" and "poor supply and access to electricity" that clearly mentioned by the participants ( also check the part of "lack of learning space").

 These obstacles, more or less, actually also happened everywhere and many reports offer solution or at least ‘shortcut ^4^^,^^5^. ^^The data these investigators revealed showed us how they tried their best to adjust and even modify everything in order to make sure that at least minimum education delivered.

 One of the shortcomings that also needs to be revealed is how the government react and support the medical institutions, because many of the roots of the problems are far beyond the reach of students, lecturers, academic staffs and institutions, but actually within the jurisdiction of the government.

 To my opinion this article deserve compliment and worth to be read and studied in more depth; and I am grateful that this journal, in its high moral values, accepted this simple qualitative article to open our minds about the importance of support and equality for those in need.

 References

1. Sawatsky AP, Ratelle JT, Beckman TJ. Qualitative Research Methods in Medical Education. Anesthesiology. 2019 Jul;131(1):14-22. https://doi.org/10.1097/ALN.0000000000002728.
2. Rastogi A, Bansal A, Keshan P, Jindal A, Prakash A, Kumar V. Medical education in post-pandemic times: Online or offline mode of learning? J Family Med Prim Care. 2022 Sep;11(9):5375-5386. https://doi.org/10.4103/jfmpc.jfmpc_2305_21.
3. U.S Department of State. 2023 Investment Climate Statements: Zambia. Downloaded from https://www.state.gov/reports/2023-investment-climate-statements/zambia/
4. Daroedono E, Siagian FE, Alfarabi M, Cing JM, Arodes ES, Sirait RH, et al. The impact of COVID-19 on medical education: our students perception on the practice of long distance learning. Int J Community Med Public Health 2020;7:2790-6. https://doi.org/10.18203/2394-6040.ijcmph20202545
5. Grafton-Clarke C, Uraiby H, Gordon M, Clarke N, Rees E, Park S, et al. Pivot to online learning for adapting or continuing workplace-based clinical learning in medical education following the COVID-19 pandemic: A BEME systematic review: BEME Guide No. 70. Med Teach. 2022 Mar;44(3):227-243. https://doi.org/10.1080/0142159X.2021.1992372.

Is the work clearly and accurately presented and does it cite the current literature?

Yes

If applicable, is the statistical analysis and its interpretation appropriate?

I cannot comment. A qualified statistician is required.

Are all the source data underlying the results available to ensure full reproducibility?

Yes

Is the study design appropriate and is the work technically sound?

Yes

Are the conclusions drawn adequately supported by the results?

Yes

Are sufficient details of methods and analysis provided to allow replication by others?

Yes

Reviewer Expertise:

Parasitology, Mycology, Medical Education

I confirm that I have read this submission and believe that I have an appropriate level of expertise to confirm that it is of an acceptable scientific standard.

## References

[1] : Qualitative Research Methods in Medical Education. *Anesthesiology* .2019;131(1) : 10.1097/ALN.0000000000002728 14-22 10.1097/ALN.0000000000002728 31045898

[2] : Medical education in post-pandemic times: Online or offline mode of learning?. *J Family Med Prim Care* .2022;11(9) : 10.4103/jfmpc.jfmpc_2305_21 5375-5386 10.4103/jfmpc.jfmpc_2305_21 36505568 PMC9731027

[3] : Investment Climate Statements: Zambia.2023; Reference source

[4] : The impact of COVID-19 on medical education: our students perception on the practice of long distance learning. *International Journal Of Community Medicine And Public Health* .2020;7(7) : 10.18203/2394-6040.ijcmph20202545 10.18203/2394-6040.ijcmph20202545

[5] : Pivot to online learning for adapting or continuing workplace-based clinical learning in medical education following the COVID-19 pandemic: A BEME systematic review: BEME Guide No. 70. *Med Teach* .2022;44(3) : 10.1080/0142159X.2021.1992372 227-243 10.1080/0142159X.2021.1992372 34689692
